# Influences of Molecular Weights on Physicochemical and Biological Properties of Collagen-Alginate Scaffolds

**DOI:** 10.3390/md19020085

**Published:** 2021-02-02

**Authors:** Truc Cong Ho, Jin-Seok Park, Sung-Yeoul Kim, Hoyeol Lee, Ju-Sop Lim, Shin-Jun Kim, Mi-Hee Choi, Seung Yun Nam, Byung-Soo Chun

**Affiliations:** 1 Institute of Food Science, Pukyong National University, 45 Yongso-ro, Nam-gu, Busan 48513, Korea; hocongtruc@pukyong.ac.kr; 2 Department of Food Science and Technology, Pukyong National University, 45 Yongso-ro, Nam-gu, Busan 48513, Korea; jin1931@pukyong.ac.kr (J.-S.P.); kim41409114@pukyong.ac.kr (S.-Y.K.); 3 Industry 4.0 Convergence Bionics Engineering, Pukyong National University, Busan 48513, Korea; lhy950@pukyong.ac.kr; 4 PL MICROMED Co., Ltd., 1F, 15-5, Yangju 3-gil, Yangsan-si, Gyeongsangnam-do 50620, Korea; casseur1@plmicromed.com (J.-S.L.); shinjun@plmicromed.com (S.-J.K.); mhchoi28@plmicromed.com (M.-H.C.); 5 Department of Biomedical Engineering, Pukyong National University, Busan 48513, Korea; synam@pknu.ac.kr

**Keywords:** low molecular weight collagen, subcritical water modified alginate, scaffolds, physicochemical properties, coagulant activity, antioxidant capacity

## Abstract

For tissue engineering applications, biodegradable scaffolds containing high molecular weights (MW) of collagen and sodium alginate have been developed and characterized. However, the properties of low MW collagen-based scaffolds have not been studied in previous research. This work examined the distinctive properties of low MW collagen-based scaffolds with alginate unmodified and modified by subcritical water. Besides, we developed a facile method to cross-link water-soluble scaffolds using glutaraldehyde in an aqueous ethanol solution. The prepared cross-linked scaffolds showed good structural properties with high porosity (~93%) and high cross-linking degree (50–60%). Compared with collagen (6000 Da)-based scaffolds, collagen (25,000 Da)-based scaffolds exhibited higher stability against collagenase degradation and lower weight loss in phosphate buffer pH 7.4. Collagen (25,000 Da)-based scaffolds with modified alginate tended to improve antioxidant capacity compared with scaffolds containing unmodified alginate. Interestingly, in vitro coagulant activity assay demonstrated that collagen (25,000 Da)-based scaffolds with modified alginate (C25-A63 and C25-A21) significantly reduced the clotting time of human plasma compared with scaffolds consisting of unmodified alginate. Although some further investigations need to be done, collagen (25,000 Da)-based scaffolds with modified alginate should be considered as a potential candidate for tissue engineering applications.

## 1. Introduction

Collagen is the most abundant structural protein in the body. Until now, 26 types of collagen have been classified, and they are divided into eight families depending on their structure, chain bonding, and position in the body [1]. Collagen type I is a major form of collagen in higher vertebrates. It is distributed mainly in the skin, muscle, bone, and viscera. Lower vertebrates like fish also have type I collagen that is mostly seen in the scale, skin, bone, and swim bladders [2]. Collagen molecules possess distinctive properties such as biodegradation, weak antigen expression, biocompatibility, and self-assembling fibril formation [3]. Due to its versatility, collagen has been widely used in the food, cosmetic, and healthcare industries. Recently, some researchers have demonstrated that collagen can be used as a novel protein-based material in biomedical applications [4,5,6,7]. Collagen has been used alone [8] or in combination with other biopolymers such as chitosan and alginate to produce degradable scaffolds for diabetic wound healing [7] and skin tissue engineering [4,5,6,9,10]. However, collagen having a high molecular weight (MW) shows lower bioactivities than collagen peptides that have low MW [11]. Besides, individual biopolymers do not possess desired properties such as biodegradation and mechanical toughness. Fortunately, cross-linked biopolymers could possess such properties [12].

Alginate is a heteropolysaccharide that consists of two-unit monosaccharides: d-mannuronic acid (M) and l-guluronic acid (G) [13]. This biopolymer has been widely used in a variety of industries such as food, cosmetics, and healthcare. Due to the physicochemical properties of alginate, which change according to its MW, alginate is depolymerized using hydrogen peroxide [14] and periodate [15]. According to Li et al. (2009), the depolymerized alginate can be used in biodegradable tissue engineering and drug delivery because it shows a faster degradation rate compared with that of unmodified alginate [14]. However, the depolymerization process of alginate uses many highly concentrated chemicals that might result in some risks to human health [16]. Fortunately, alginate was also depolymerized using subcritical water at 180 °C‒250 °C [13]. Subcritical water is water at a temperature above 100 °C and below 374 °C and pressure high enough to keep it in a liquid state [17]. Under subcritical water conditions, the depolymerization sequence undoubtedly occurs and could allow the modification of alginate MW distributions [13]. There has been one study about the influences of different MW of oxidized alginate using sodium periodate cross-linked with collagen fiber [15]. However, the effects of low MW of both collagen and alginate on the properties of collagen-based scaffolds have not been examined.

Most of the distinctive properties in extracted collagen are lost or weaker than those in its natural state due to the influences of extraction processes. The loss of the original characters of collagen causes significant restraint in its biotechnological applications [3]. To overcome these problems, cross-linking treatment is necessary to link collagen molecules or collagen with other biopolymers in collagen-based biomaterials. Chemical methods and physical methods are two types of cross-linking methods recently used to improve the properties of collagen-based scaffolds [9]. In chemical methods, some cross-linkers have been commonly used to enhance the physical properties of collagen-based biomaterials such as 1-ethyl-3-(3-dimethylaminopropyl) carbodiimide/N-hydroxysuccinimide (EDC/NHS) [5,18], glutaraldehyde (GTA) [9,19], and genipin [20]. Among cross-linking agents, glutaraldehyde is inexpensive and predominantly employed since it can react with functional groups in both proteins and carbohydrates and can provide materials with substantial improvement in tensile properties [21]. Moreover, it is reported that GTA is noncytotoxic at a concentration of up to 8% [21,22].

From the literature review, in the present work, we used collagen (6000 and 25,000 Da) and alginate unmodified and modified by subcritical water at 110, 120, and 130 °C for producing composite scaffolds with a hypothesis that they could be highly flexible and bioactive but still could maintain distinctive properties of biodegradable scaffolds. The water-soluble scaffolds were cross-linked by GTA in an aqueous ethanol solution. Finally, the scaffolds were characterized in terms of Fourier-transform infrared spectroscopy (FTIR), microstructure (SEM), porosity, weight loss, water retention, mechanical properties, enzymatic degradation, antioxidant activity, and in vitro blood coagulant capacity.

## 2. Results

### 2.1. Molecular Weight of Collagen and Alginate

MW of collagen and subcritical water modified sodium alginate were determined using gel permeation chromatography (GPC) and the results were presented in Table 1. MW of original alginate ranged from 32,000 to 250,000 Da, and its MW decreased to 125,000, 63,000, and 21,000 Da, as the thermal treatment process increased from 110 °C, 120 °C, and 130 °C, respectively.

### 2.2. Degree of Cross-Linking

**Figure 1:**
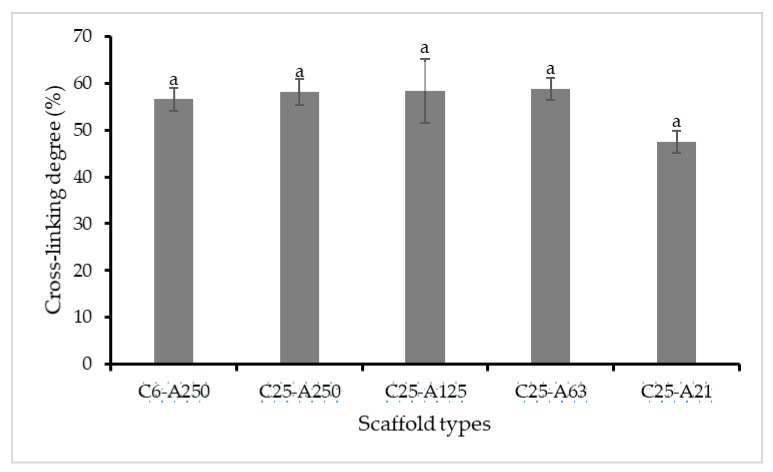
Cross-linking degree of collagen-alginate scaffolds. C6-A250—scaffold contains collagen (6000 Da) and alginate (32,000–250,000 Da); C25-A250—scaffold contains collagen (25,000 Da) and alginate (32,000–250,000 Da); C25-A125—scaffold contains collagen (25,000 Da) and alginate (125,000 Da); C25-A63—scaffold contains collagen (25,000 Da) and alginate (63,000 Da); C25-A21—scaffold contains collagen (25,000 Da) and alginate (21,000 Da). Letters “a” indicate that cross-linking degree of the scaffolds are not significantly different (*p* ≤ 0.05).

Cross-linking degree is related to free amine groups and they are inversely proportional. Glutaraldehyde (GTA) can cross-link collagen molecules via the reaction between free amine groups of lysine and hydroxyl-lysine amino acid residues of the polypeptide chains with the aldehyde groups [19] and can react with functional groups in both proteins and carbohydrates [21]. The cross-linking degree of scaffolds that are made from collagen (6000 and 25,000 Da) and alginate with and without hydrothermal treatment was determined and presented in Figure 1. The results showed that there was a comparison in cross-linking degree of scaffolds including C6-A250, C25-A250, C25-A125, and C25-A63 with approximately 58%. However, sponge C25-A21 showed a slight decrease although the difference was not statistically significant.

### 2.3. FTIR Analysis

**Figure 2:**
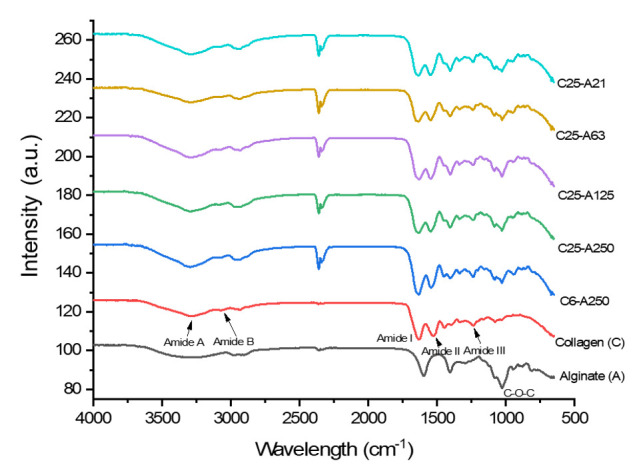
FTIR spectra of pure materials and scaffolds. C6-A250—scaffold contains collagen (6000 Da) and alginate (32,000–250,000 Da); C25-A250—scaffold contains collagen (25,000 Da) and alginate (32,000–250,000 Da); C25-A125—scaffold contains collagen (25,000 Da) and alginate (125,000 Da); C25-A63—scaffold contains collagen (25,000 Da) and alginate (63,000 Da); C25-A21—scaffold contains collagen (25,000 Da) and alginate (21,000 Da).

Fourier-transform infrared spectroscopy (FTIR) spectra and specific wavelengths of functional groups of pure collagen, alginate, and scaffolds (Figure 2 and Table 2) confirmed the homogeneity and changes in the chemical structure of sponges. For alginate, bands at wavelengths approximately 1600 and 1404 cm^−1^ are related to asymmetric and symmetric stretching vibration peaks of carboxylate salt groups, whereas a band at 1022 cm^−1^ is typically for the C‒O‒C stretching of glycosidic bonds [5,23]. In the spectra of collagen, there are five characteristic bands including amide A, amide B, amide I, amide II, and amide III. Specifically, the peak at wavelength 3282 cm^−1^ is typically for N‒H stretching vibrations representing amide A. The next band at approximately 3057 cm^−1^ is typically for C‒H stretching of amide B, whereas the band at 1627 cm^−1^ is characteristic for the stretching vibrations of C=O couples to N‒H of amide I. The band at 1525 cm^−1^ is represented for stretching vibrations of N‒H couple to C‒N of amide II. A typical band of amide III is also observed at wavelength approximately 1236 cm^−1^. The FTIR peaks of scaffolds are the combination of collagen and alginate peaks that showed all amide bands of collagen and glycosidic bonds C‒O‒C of alginate, indicating that composite scaffolds were successfully fabricated.

### 2.4. Microstructure Analysis (SEM) of the Scaffolds

**Figure 3:**
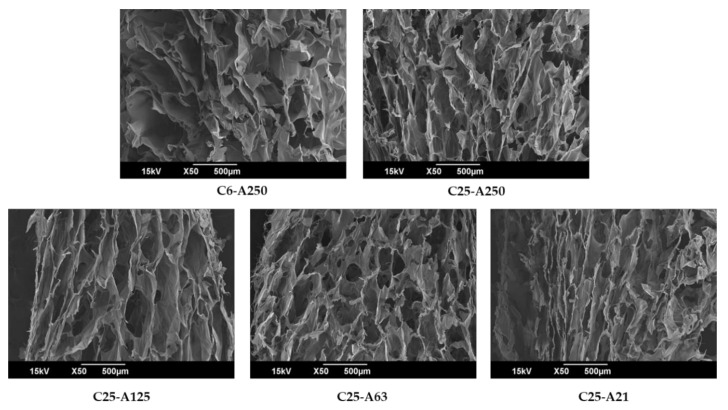
Scanning electron microscope of pure materials and scaffolds. C6-A250—scaffold contains collagen (6000 Da) and alginate (32,000–250,000 Da); C25-A250—scaffold contains collagen (25,000 Da) and alginate (32,000–250,000 Da); C25-A125—scaffold contains collagen (25,000 Da) and alginate (125,000 Da); C25-A63—scaffold contains collagen (25,000 Da) and alginate (63,000 Da); C25-A21—scaffold contains collagen (25,000 Da) and alginate (21,000 Da).

The porous structure of scaffolds plays a crucial role that can have a significant impact on other properties such as cell attachment, proliferation, neovascularization, and diffusion of oxygen and necessary nutrients during the healing process. Hence, ideal scaffolds that can be applied in tissue engineering should have a mean pore size between 100 and 200 µm [5]. Therefore, in this study, cross-section images of the scaffolds were investigated by scanning electron microscope (SEM) to confirm their porous organization, and the results are presented in Figure 3. Cross-section images showed that the pore size of scaffolds is less than 500 µm, and there were some differences between them. C6-A250 exhibited ununiform and less interconnected porous architecture, whereas the remaining scaffolds were quite uniform and well interconnected. C25-A125 has the largest pores; C25-A63 had a smaller pore size (100–200 µm) and relatively round shape, whereas pores of C25-A250 and C25-A21 scaffolds had long, narrow shape and were less interconnected as compared with those of C25-A125 and C25-A63 scaffolds.

### 2.5. Porosity, Density, and Compressive Properties of the Scaffolds

**Figure 4:**
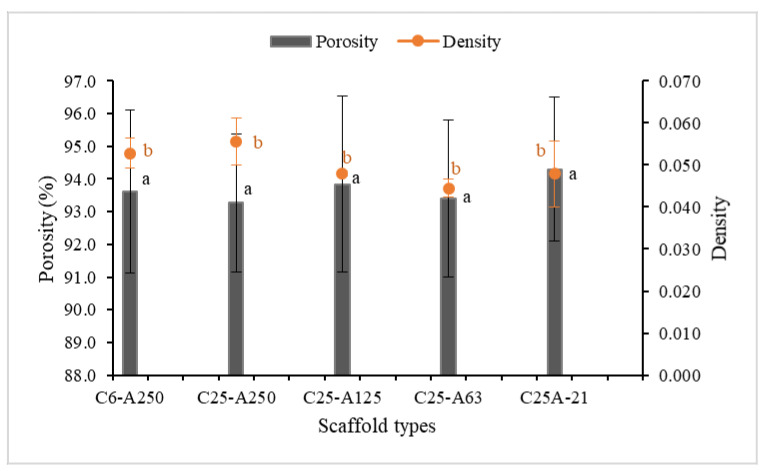
Porosity and density of scaffolds. C6-A250—scaffold contains collagen (6000 Da) and alginate (32,000–250,000 Da); C25-A250—scaffold contains collagen (25,000 Da) and alginate (32,000–250,000 Da); C25-A125—scaffold contains collagen (25,000 Da) and alginate (125,000 Da); C25-A63—scaffold contains collagen (25,000 Da) and alginate (63,000 Da); C25-A21—scaffold contains collagen (25,000 Da) and alginate (21,000 Da). Letters “a” indicate that the porosity of all the scaffolds are not significantly different (*p* ≤ 0.05); letters “b” indicate that the density of all scaffolds are not significantly different (*p* ≤ 0.05).

Porosity is also one of the important parameters of scaffolds that are applied in tissue engineering and should be greater than 90% [5]. In this study, the porosity of all scaffolds (Figure 4) was approximately 93%, and there was no significant difference between them. In addition, their density was not statistically different and ranged from 0.44 to 0.56 mg/mm^3^.

### 2.6. Tensile and Compressive Properties

**Figure 5:**
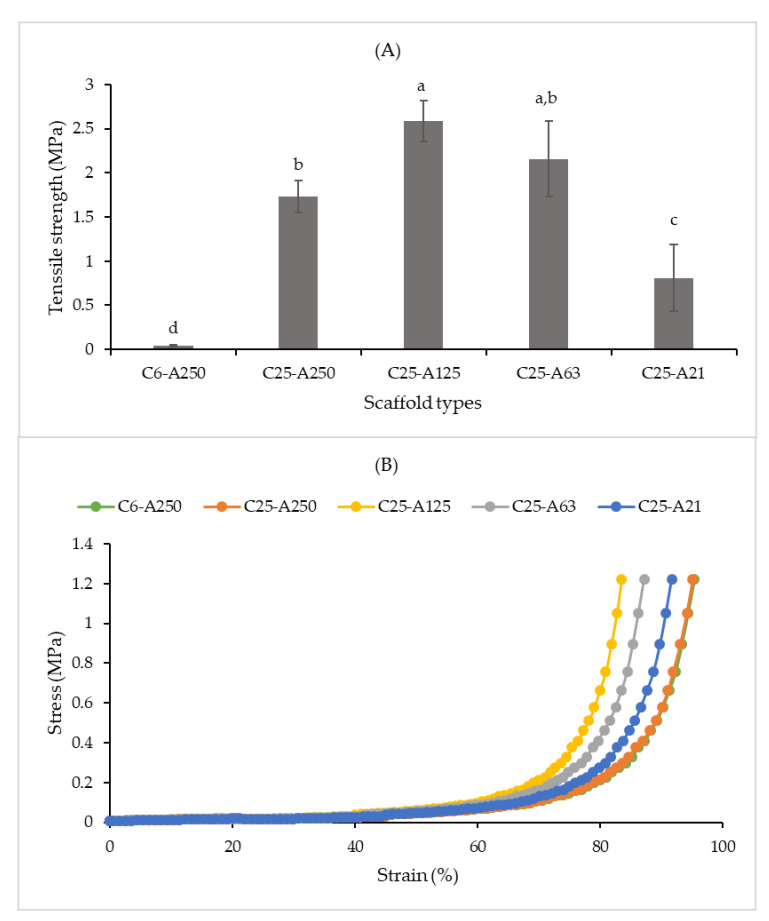
Tensile strength (**A**) and compressive property (**B**). C6-A250—scaffold contains collagen (6000 Da) and alginate (32,000–250,000 Da); C25-A250—scaffold contains collagen (25,000 Da) and alginate (32,000–250,000 Da); C25-A125—scaffold contains collagen (25,000 Da) and alginate (125,000 Da); C25-A63—scaffold contains collagen (25,000 Da) and alginate (63,000 Da); C25-A21—scaffold contains collagen (25,000 Da) and alginate (21,000 Da). Different letters (a–d) in (**A**) indicate that there are significant differences between scaffolds (*p* ≤ 0.05).

The tensile and stress–strain curves of the composite scaffolds are presented in Figure 5. The tensile (Figure 5A) showed that the C6-A250 scaffold had the lowest tensile strength with approximately 0.04 MPa. The hardness of C25-A125 and C25-A63 was significantly higher than that of the C25-A21 scaffold. For compressive property, all scaffolds are highly flexible, and they are comparable with the results from a previous study [24]. C6-A250 and C25-A250 scaffolds were the most flexible ones, whereas the remaining scaffolds showed a lower level of flexibility.

### 2.7. Weight Loss and Water Retention Capacity

**Figure 6:**
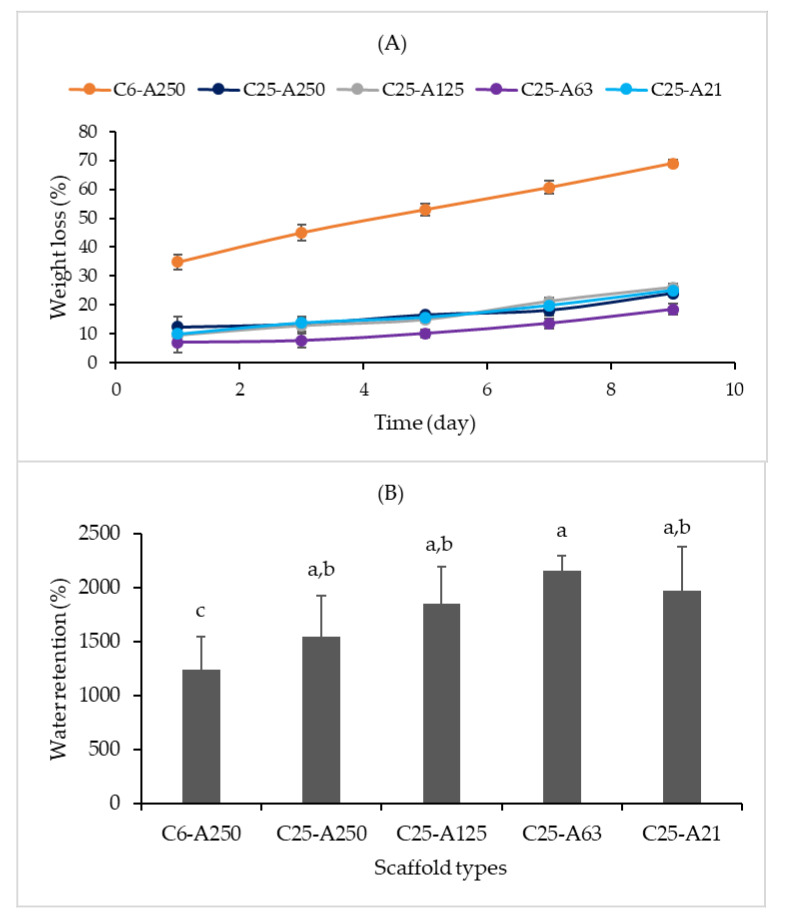
Weight loss and water retention capacity of scaffolds. (**A**)—Weight loss; (**B**) —Water retention capacity. C6-A250—scaffold contains collagen (6000 Da) and alginate (32,000–250,000 Da); C25-A250—scaffold contains collagen (25,000 Da) and alginate (32,000–250,000 Da); C25-A125—scaffold contains collagen (25,000 Da) and alginate (125,000 Da); C25-A63—scaffold contains collagen (25,000 Da) and alginate (63,000 Da); C25-A21—scaffold contains collagen (25,000 Da) and alginate (21,000 Da). Different letters (a–c) in (B) indicate that there are significant differences between scaffolds (*p* ≤ 0.05).

Weight loss and water retention properties of composite scaffolds are presented in Figure 6. C6-A250 scaffold had the highest weight loss ratio (Figure 6A) with 35% after the first day and 70% after nine days in phosphate buffer at 37 °C. On the other hand, the remaining scaffolds were more stable under the same conditions when their weight loss was less than 10% after one day incubation and less than 30% after nine days. The water retention capacity of scaffolds (Figure 6B) was quite high and ranged from 1200% to 2000%. Among those, C6-A250 has the lowest water retention with approximately 1240%, and it was significantly different from the C25-A63 scaffold. On the other hand, C25-A63 had the highest water retention capacity with approximately 2157%. Although the difference between C25-A250, C25-A125, C25-A63, and C25-A21 was not statistically significant, there was a trend that the water retention capacity of scaffolds got a peak at C25-A63 (Figure 6B).

### 2.8. Enzymatic Degradation

**Figure 7:**
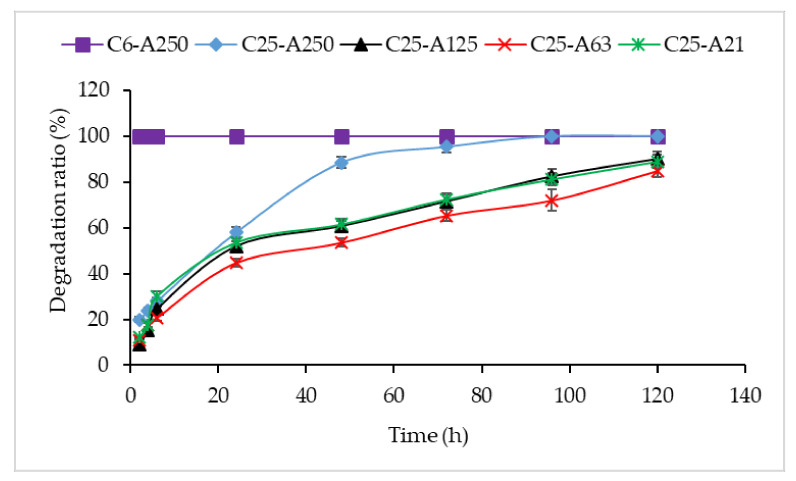
Enzymatic degradation of scaffolds. C6-A250—scaffold contains collagen (6000 Da) and alginate (32,000–250,000 Da); C25-A250—scaffold contains collagen (25,000 Da) and alginate (32,000–250,000 Da); C25-A125—scaffold contains collagen (25,000 Da) and alginate (125,000 Da); C25-A63—scaffold contains collagen (25,000 Da) and alginate (63,000 Da); C25-A21—scaffold contains collagen (25,000 Da) and alginate (21,000 Da).

Enzymatic degradation of scaffolds in PB pH 7.4 containing collagenase (60 µg/mL) for 5 days was carried out at 37 °C, and the results were presented in Figure 7. The C6-A250 scaffold was fully degraded after incubating 2 h in collagenase solution. As compared with C6-A250, C25-A250 was more resisted against collagenase when its degradation ratio was approximately 20% after 2 h and remained after 72 h before completely degraded after 96 h. The remaining scaffolds including C25-A125, C25-A63, and C25-A21 were quite stable after incubating for 120 h. In detail, the degradation ratio of these three scaffolds was around 10% after 2 h of incubation, and the ratio was less than 30% after 6 h. More than 50% of scaffolds was degraded after 24 h in scaffolds C25-A125 and C25-A21; however, the ratio in C25-A63 was only 45%. Overall, C25-A63 was more stable than other scaffolds in the enzymatic degradation process.

### 2.9. Antioxidant Capacity

**Figure 8:**
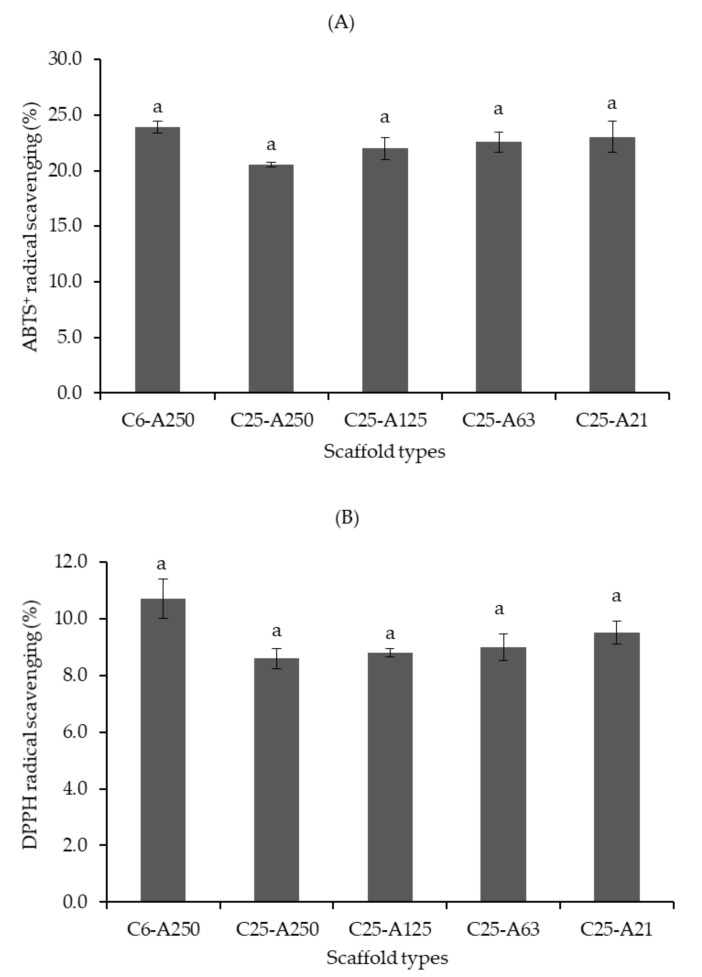
Antioxidant capacity of collagen-alginate scaffolds: (**A**)—ABTS^+^ radical scavenging and (**B**)—DPPH radical scavenging. C6-A250—scaffold contains collagen (6000 Da) and alginate (32,000–250,000 Da); C25-A250—scaffold contains collagen (25,000 Da) and alginate (32,000–250,000 Da); C25-A125—scaffold contains collagen (25,000 Da) and alginate (125,000 Da); C25-A63—scaffold contains collagen (25,000 Da) and alginate (63,000 Da); C25-A21—scaffold contains collagen (25,000 Da) and alginate (21,000 Da). Letters “a” indicate that there is no significant difference between scaffolds (*p* ≤ 0.05).

The antioxidant capacity of uncross-linked scaffolds (Figure 8) was investigated using two common assays, including ABTS^+^ (A) and DPPH (B) radical scavenging. There was no significant difference in scavenging capacity between scaffolds. However, there was a trend that C6-A250 scaffolds have the highest percentage scavenging with approximately 23% and 11% for ABTS^+^ and DPPH, respectively. Among the remaining scaffolds, there was another trend that radical scavenging activity of scaffolds tended to increase with the decrease in MW of alginate or the increase in temperature that was used to depolymerize alginate polymers. DPPH percentage scavenging of C25-A250, C25-A125, C25-A63, and C25-A21 was 8.6% ± 0.4%, 8.8% ± 0.2%, 9.0% ± 0.5%, and 9.5% ± 0.4%, respectively, and ABTS^+^ percentage scavenging of these samples was 20.6 ± 0.5, 22.0 ± 0.2, 22.6 ± 0.9, and 23.0 ± 1.4, respectively.

### 2.10. In Vivo Blood Coagulant Assay

**Figure 9:**
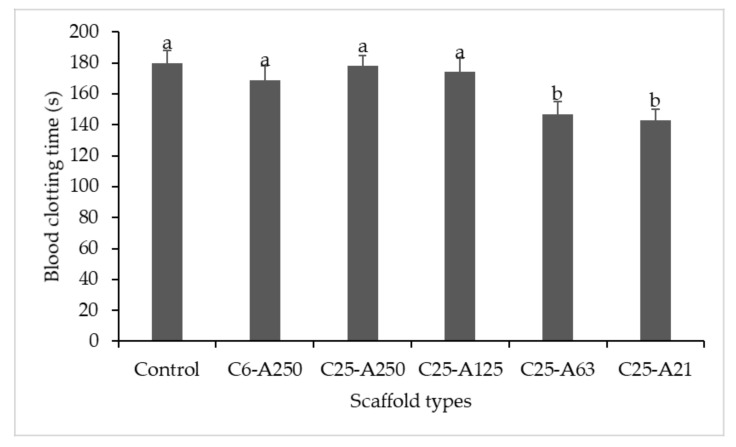
Blood coagulant activity of sponges. C6-A250—scaffold contains collagen (6000 Da) and alginate (32,000–250,000 Da); C25-A250—scaffold contains collagen (25,000 Da) and alginate (32,000–250,000 Da); C25-A125—scaffold contains collagen (25,000 Da) and alginate (125,000 Da); C25-A63—scaffold contains collagen (25,000 Da) and alginate (63,000 Da); C25-A21—scaffold contains collagen (25,000 Da) and alginate (21,000 Da). Different letters (a–b) indicate that there are significant differences between scaffolds (*p* ≤ 0.05).

In vitro blood coagulation activity of collagen–alginate against human plasma of scaffolds was examined, and the result is presented in Figure 9. In this test, deionized water was used as the control test to determine the clotting time of human plasma. The time for plasma clot form of the control was recorded with approximately 180 ± 8.4 s and was similar to that of C25-A250 (178 ± 7.3 s). For the C6-A250 scaffold, the clotting time (169 ± 9.2 s) was reduced 11 s as compared with the control and 9 s shorter than C25-A250 scaffolds. The remaining scaffolds that contained modified alginate tended to improve the coagulant activity and increased with the increase in the treating temperature of subcritical water from 110 °C to 130 °C. The scaffolds C25-A125 reduced the blood clotting time by 6 s as compared with the control one. Especially, scaffolds C25-A63 and C25-A21 significantly reduced the clotting time (33 ± 8.6 s and 37 ± 8.1 s), respectively, in comparison with the control.

## 3. Discussion

In this study, we hypothesized that scaffolds made from the low MW of collagen as well as alginate might have a soft structure and perform good functionalities. However, they remain distinctive properties that can be suitable in wound-stressing applications. One of those is flexibility property that is beneficial for wound stressing since it can have close contact with the wounded surface [24]. For that reason, two different MWs of collagen (6000 Da and 25,000 Da) were employed. Besides, MW of alginate was modified using subcritical water at temperatures 110 °C, 120 °C, and 130 °C. This is the first study on the use of subcritical water modified alginate that was then applied in tissue engineering. According to Aida et al. (2010), hydrothermal treatments are a promising method in the modification of the structure of alginate molecules. Under hydrothermal conditions, alginate is decomposed at the glycosidic bonds through an acid hydrolysis pathway [13]. When the temperature increased from 110 °C to 130 °C, the molecular weight of alginate decreased from 125,000 Da to 21,000 Da, whereas the original alginate has MW at around between 32,000 and 250,000 Da.

Scaffolds fabricated by low MW collagen and alginate are water-soluble; therefore, they need to be cross-linked to make them more stable in aqueous conditions. In our work, some screening experiments about the use of cross-linking agents including EDC, formaldehyde, and GTA in 95% ethanol were carefully carried out. The results showed that formaldehyde and GTA had better cross-linking ability than EDC; however, formaldehyde is known as more toxic than GTA. Therefore, in this study, we developed a simple technique to cross-link water-soluble collagen-based biomaterials using GTA in 95% ethanol. Results indicated that the cross-linking degree was found to be between 48% and 60%. This result was similar to that reported in a previous study when tilapia skin collagen sponge has approximately 60% after cross-linking by 0.625% GTA [25]. According to Choi et al. (1999), gelatin and alginate could be cross-linked via carboxyl groups that are rich in these two polymers when EDC is used as a cross-linker [26]. Between collagen and other proteins, GTA reacts with the free amine group of lysine or hydroxyl-lysine amino acid residues of the polypeptide chains to form Schiff base intermediates [19]. Cross-linking degree tended to decrease when collagen combined with alginate modified at 130 °C. This might be due to more alginate molecules that are produced under subcritical water hydrolysis cross-linked with GTA via their –OH groups. Therefore, there was an increase in the number of GTA molecules cross-linked with alginate, leading to the decrease in the number of GTA molecules cross-linked with collagen. In this case, the degree of cross-link between alginate with GTA cannot be tested by TNBS method. Fortunately, cross-linked alginate could also cross-link with collagen. Consequently, although the degree of crosslink between collagen and GTA slightly decrease (Figure 1), the total cross-linking degree might not be much different. As we expected, low MW of collagen and alginate fabricated scaffolds cross-linked with GTA possess a flexible structure. According to L. Sun et al. (2018), GTA can cross-link collagen molecules via covalent bonds between its carbonyl groups by removing two oxygen atoms and amine groups of collagen [25]. However, intramolecular forces between collagen molecules may be weaker than intermolecular forces inside them. This might be the reason that the C6-A250 scaffold possessed a soft structure than those obtained from collagen 25,000 Da. SEM image of C6-A250 (Figure 3) also confirmed this phenomenon when its porous structure had some physical damages that are not found in the remaining scaffolds. Due to the weakness in its structure, the damages to the scaffold might be caused either by themselves or by the sample preparation step for SEM analysis.

In our study, the porosity of scaffolds was determined at approximately 93%. This value was found to be around 90% in previous work that also studied similar materials [5]. Considering that, the MW of the biopolymers are higher than those used in the present study. Meanwhile, according to some studies, the porosity of high MW collagen alone scaffolds is reported to be around 97% [8,25]. Therefore, it can be concluded that: first, low MW collagen-based scaffolds might have higher porosity than high MW collagen-based scaffolds; second, collagen alone scaffolds might have higher porosity than collagen-based biomaterials.

Water retention capacity can be influenced by the porous structure of scaffolds. The results from this study demonstrated that C25-A63 scaffolds that had round, small, and interconnected porous structure held the highest water retention ratio, whereas the water retention ratio of scaffolds with a large pore size (C25-A110) or less interconnected (C25-A250 and C25-A21) tended to decrease.

Weight loss and enzymatic degradation ratio are also two crucial properties that are significantly affected by the MW of collagen used. Although cross-linking degree in all scaffolds was similar, the high weight loss ratio of C6-A250 as compared with C25-A250 might be caused by its low MW. According to a previous report, low MW collagen is more soluble in aqueous conditions due to the exposure of polar amino acid residues capable of binding water through hydrogen bonds [27]. Consequently, the aqueous solution containing collagenase can quickly accelerate the decomposition of low MW collagen. There are some ways for collagenase to cleavage collagen molecules: First, unwound collagen α chains, but not triple-helical structure, may be a prerequisite for collagenase to cleave interstitial collagen, and second, collagenase could cleave single α chain during collagenolysis [28]. The resistance against collagenase degradation of scaffolds C25-As might be due to the consistency of high MW collagen, which has less unwound α chains and less exposed polar amino acid. Besides, alginate MW could affect the stability of composite scaffolds against enzymatic degradation. Moderate MW of alginate had better protection for the collagen biodegradation process since they might well cover the surface of collagen structure than unmodified alginate and low MW one.

Although there was no significant difference in antioxidant activity between composite scaffolds, there was a trend that the C6-A250 scaffold showed the highest ABTS^+^ and DPPH radical scavenging activity as compared with the remaining scaffolds. It can be explained that low MW collagen might contain more electron donors that could scavenge free radicals, leading to the formation of stable products [29]. This result once again confirms that low MW collagen is presumed to have better bioavailability than larger peptides and parent proteins [11]. Another trend is that low MW alginate could also assist in the improvement of the antioxidant capacity. Due to the cleavage of glycosidic bonds under subcritical water hydrolysis, more functional groups such as hydroxyl, carbonyl, and carboxyl groups are produced, leading to an increase in the electron donation process for the stabilization of free radicals [30]. Composite scaffolds also showed capability in the reduction of blood coagulation time. The results in this study were consistent with those of previous reports [8,25]. Surprisingly, the scaffolds containing alginate modified at 120 °C and 130 °C (C25-A63 and C25-A21) significantly reduced blood clotting time as compared with the control test. This may be due to the synergistic effects of the low MW of both collagen and alginate rather than a single compound. More investigation is needed to confirm the mechanism of this effect. Although the biological properties were determined using uncross-linked scaffolds since they are water-soluble, the cross-linked scaffolds might also possess such promising properties. Therefore, further examinations of the scaffolds on animals should be also considered in future studies.

## 4. Materials and Methods

### 4.1. Chemicals

Collagen (6000 and 25,000 Da) was kindly donated by PL Micromed Co., Ltd. (Gyeongsangnam-do, Korea). Alginate with MW ranging from 32,000 to 250,000 Da was obtained from Duksan Company (Gyeonggi-do, Korea). Glutaraldehyde was purchased from Junsei Chemical Co., Ltd. (Tokyo, Japan). The aPTT reagent and human plasma were purchased from Thermo Fisher Scientific, Pittsburgh, PA, USA. Other chemicals were of analytical grade.

### 4.2. Methods

#### 4.2.1. Modification of Alginate Molecular Size Using Subcritical Water

The modification of alginate was done using a subcritical water hydrolysis system equipped with a 1 L reactor. Alginate (3%) was well suspended in distilled water to assist the hydrolysis before introducing into the reactor. CO_2_ was used to pressurize the system and to maintain the pressure at 30 bar during the process. Alginate was modified at three different temperatures (110 °C, 120 °C, and 130 °C) under subcritical water conditions with a fixed reaction time and pressure (30 min and 30 bar, respectively). Because the initial alginate solution possesses high viscosity, the stirring speed needs to be high enough to create a well-mixed solution. Therefore, a stirring speed at 700 rpm was set to assist the depolymerization process well homogeneous. Furthermore, the modified alginate solution was stored at 4 °C for further experiments.

#### 4.2.2. Gel Permeation Chromatography

MW of collagen and subcritical water modified alginate were determined using GPC. First, samples were dissolved in deionized water with a concentration of 3 mg mL^−1^. The solution was then filtered through a 0.45 µm pore size Nylon filter and degassed. In the GPC program, the injection volume was kept at 100 µL, and 0.1 M of NaNO_3_ was employed as a developing solvent with a flow rate of 1 mL min^−1^. The MW of modified alginate and its distribution were analyzed at 40 °C using Tosoh EcoSEC HLC-8320 GPC equipped (Tosoh Bioscience, Hesse, Germany) with RI detector and TSKgel guard PW × l + 2 × TSKgel MPW × l + TSKgel G2500PW × l (7.8 × 300 mm) column. The determination of the MW of modified alginate and collagen was based on the calibration curve of the pullulan standards (180 to 642 × 10^3^ g mol^−1^) and polyethylene glycol/polyethylene oxide standards (600 to 122,000 Da), respectively. The results were analyzed using the EcoSEC software (Tosoh, version No. 1.11, Hesse, Germany).

#### 4.2.3. Preparation of Collagen–Alginate Scaffolds

Collagen with different MWs accurately measured and dissolved in 0.02 M acetic acid under stirring heating at 40 °C to form a solution of 0.8% of collagen. The solution was then neutralized using 2 M NaOH in phosphate buffer. Original alginate was dissolved in deionized water under stirring and heating at 45 °C for 30 min to get the final concentration of 3% (similar to the concentration of modified alginate used in Section 4.2.1). Collagen solutions were well mixed with modified and original alginate solutions separately using a stirrer at 250 rpm, at room temperature to attain a ratio of collagen/alginate, 7:3 (*w/w*). The mixtures were then kept at 4 °C for 2 days under stirring at 130 rpm. The resulting mixtures were then centrifuged at 3000 rpm for 15 min to eliminate accumulated air bubbles before pouring into cell culture plates with a volume of 40 mL/plate. Next, plates containing collagen–alginate blend were kept at 25 °C for 12 h allowing collagen gelation. The incubated blends were subsequently frozen at −20 °C and −80 °C for 12 h per each step. The frozen blends were freeze-dried using a freeze dryer HyperCool HC4110 (GYROEN Co., Ltd., Gyeonggi-do, Korea). The lyophilized 3D scaffolds were then cross-linked using 0.5% GTA in 95% ethanol for 1.5 h at room temperature before cleaning with absolute ethanol five times and freeze-dried for 12 h. The cross-linked scaffolds were stored at room temperature in a zip bag for further characterization.

### 4.3. Characterizations of Collagen–Alginate Scaffolds

#### 4.3.1. Fourier-Transform Infrared Spectroscopy (FTIR)

Scaffolds were mixed with KBr (spectrum pure) with a ratio of approximately 1:100, *w/w*. The mixture was ground and pressed into transparent sheets. To determine the functional groups of scaffolds, FTIR spectra were determined at a resolution of 4 cm^−1^ with wavelength ranging from 400 to 4000 cm^−1^ at room temperature using Perkin Elmer, Inc. (Waltham, MA, USA), and data were analyzed using Origin 2019b software (OriginLab Corporation, Version 9.65, Northampton, MA, USA).

#### 4.3.2. Microstructure Characterization

The microstructure of scaffolds was characterized using a JEOL, JSM-6700 F, Field Emission Scanning Electron Microscopy (JEOL Ltd., Akishima, Tokyo 196-8558, Tokyo, Japan).

#### 4.3.3. Porosity and Density Assessment

The measurement of porosity and density of scaffolds was done using an ethanol infiltration method reported previously [31]. Porosity (*P*) was indirectly measured via volumes (*V_s_*) and pores (*V_p_*) of the scaffolds using the equation *P = V_p_/V_s_ × 100%*. According to this, *V_s_* was determined from the three-dimensional geometry (length, width, and height) of the scaffolds. *V_p_* was obtained from the equation *V_p_ = (W_c_ − W_o_)/ρ_e_,* where *ρ_e_* (0.789 mg mL^−1^) is ethanol density at room temperature and *W_o_* and *W_c_* are initial weight and after weight of the scaffolds, respectively, and they are determined by an ethanol infiltration method. Briefly, the weighed scaffolds (*W_o_*) were incubated in absolute ethanol at room temperature for 15 min under reduced pressure using a desiccator to remove air bubbles. The scaffolds were taken out and wiped thoroughly using a filter paper to remove ethanol on the surface and then weighed immediately (*W_c_*). The experiment was done in triplicate.

#### 4.3.4. Weight Loss

Weight loss of the scaffolds was observed for 9 days with an interval of 2 days. Briefly, precisely weighed scaffolds (*W_o_*) were incubated in 1.5 mL of phosphate buffer pH 7.4 at 37 °C in an oven. For every 2 days, incubated scaffolds were taken out, lyophilized, and weighed (*W_t_*). Subsequently, lyophilized scaffolds were again incubated in 1.5 mL of fresh phosphate buffer solution. The weight loss ratio was calculated as presented in formula 1. The experiment was carried in triplicate for each scaffold, and the results are reported as mean ± standard deviation (SD) (*n* = 3).

*W_L_* (%) = [(*W*_0_ − *W_t_*)/*W*_0_] × 100 (1)

#### 4.3.5. Tensile and Compressive Property

Scaffolds were prepared in 10 × 10 × 4 mm for compressive assessment and 20 × 5 × 4 for the tensile test. The measurements were done by using a universal testing machine LR5K Plus (LLOYD instruments, Hampshire, United Kingdom) at room temperature. The cross-head speed of both tests was set at 0.1 mm/s. In the tensile test, the experiment was progressed until failure, and in the compressive test, the compressive stress was measured at 90% strain since the scaffolds did not fracture during the compression. At least five samples per scaffold were used in both tests, and the results were expressed as mean ± SD (*n* = 5).

#### 4.3.6. Water Retention Capacity

To determine the water retention capacity of scaffolds, the weighed scaffolds (*W*_0_) were immersed into PBS (pH 7.4) at 37 °C for 24 h. Then, swollen scaffolds were taken out, carefully wiped with a tissue paper, and immediately weighed (*W*_1_). Water retention (*W_R_*) was done in triplicate and expressed as percentage (%):

*W_R_* (%) = [(*W*_1_ − *W*_0_)/*W*_0_] × 100 (2)

#### 4.3.7. Degree of Cross-Linking

The determination of the cross-linking degree in the scaffolds was done according to the method reported in the previous study [8]. Briefly, sponges (5 mg) were mixed with 1 mL of 4% NaHCO_3_ (*w/v*) for 30 min at room temperature. Then, the mixture was added with 1 mL of TNBS 0.5% before incubating at 40 °C for 2 h. Subsequently, the reaction was terminated by adding 3 mL of 6 M HCl and was then incubated at 60 °C for 90 min to terminate the reaction. After incubation, the mixture was diluted with 5 mL distilled water and cooled to room temperature before measuring at 345 nm using a Synergy HTX (Bio-Tek, Winooski, VT, USA) reader. In a blank control group, 3 mL of 6 M HCl was added before the addition of TNBS solution. The cross-linking degree was estimated using the following equation:

Cross-linking degree (%) = [1 − (*OD_c_* − *OD_b_*)/(*OD_n_* − *OD_b_*)] × 100 (3)

where *OD_c_* is the absorbance of scaffolds, *OD_b_* is the absorbance of the blank control group, and *OD_n_* is the absorbance of uncross-linked scaffolds.

#### 4.3.8. Enzymatic Degradation

The biodegradation property of scaffolds was investigated according to the method from the previous report [26]. Briefly, weighed scaffolds (*W*_0_) were incubated in 1.5 mL of 0.1 M phosphate buffer pH 7.4 containing 60 µg/mL collagenase (16 units) at 37 °C. After a fixed time, the scaffolds were taken out, washed with distilled water, lyophilized, and weighed (*W*_1_). The enzymatic degradation ratio *W_D_* (%) was determined using the following equation:

*W_D_* (%) = [(*W*_0_ − *W*_1_)/*W*_0_] × 100 (4)

#### 4.3.9. Antioxidant Capacity

ABTS^+^ and DPPH radical scavenging capacity of scaffolds was used to determine the antioxidant capacity of scaffolds. For ABTS^+^ assay, 7.4 mM ABTS^+^ and 2.6 mM potassium persulfate (K_2_S_2_O_8_) stock solutions were prepared separately. An equal volume of the two stock solutions was mixed and stand for 16 h in the absence of light at room temperature to form ABTS^+^ fresh solution. The fresh solution was diluted with methanol to get an absorbance of 0.7 at 734 nm. Then, 100 µL of diluted ABTS^+^ solution was mixed with 100 µL sample solution containing 1 mg/mL uncross-linked scaffolds in deionized water. The resulting mixture was incubated for 5 min at room temperature in a dark place. The measurement was done at 734 nm using a Synergy HTX reader (BioTek Instruments, Inc., Winooski, VT, USA). The control test was carried out in the same manner but 100 µL of methanol was used instead of 100 µL of the sample solution. The ABTS^+^ radical scavenging (%) was calculated as follows:

ABTS^+^ radical scavenging (%) = (*A_c_* − *A_s_*)/*A_c_* × 100 (5)

where *A_c_* is the absorbance of the control and *A_s_* is the absorbance of the samples.

For DPPH assay, 500 mM DPPH stock solution was diluted using methanol to obtain the absorbance value 1.4 at 517 nm using a Synergy HTX reader. Then, 100 µL diluted DPPH solution was mixed with 100 µL of a sample solution containing 1 mg/mL uncross-linked scaffolds in deionized water. The mixture was placed in the dark at room temperature for 30 min. The absorbance was measured at 517 nm. The control test was done under the same conditions using 100 µL methanol instead of 100 µL of the sample solution. DPPH radical scavenging (%) was calculated as follows:

DPPH radical scavenging (%) = (*A_c_* − *A_s_*)/*A_c_* × 100 (6)

where *A_c_* is the absorbance of the control and *A_s_* is the absorbance of samples.

#### 4.3.10. In Vitro Blood Clotting Assay

The determination of the blood clotting capacity of the sponges was examined according to the method reported by [32]. Uncross-linked sponges in deionized water (1 mg mL^−1^) was used in an activated partial thromboplastin time test (aPTT).

Partial thromboplastin with activator, calcium chloride (0.025 M), and sample solutions were pre-warmed at 37 °C in a water bath before use. For the control test, the test tube containing 33 µL deionized water was mixed with 50 µL of human plasma. The mixture was then incubated at 37 °C for 3 min before adding 50 µL of the aPTT solution and rapidly mixed. After incubating for another 3 min at 37 °C, the content was added with 50 µL of the pre-warmed CaCl_2_ solution while simultaneously starting a timer. Then, the test tube was gently tilted back and forth. The clotting time was immediately recorded as the first clot formed. For the tests of scaffolds, the test tube contained 33 µL of pre-warmed sample solution instead of deionized water. The blood clotting activity tests were performed in triplicate.

### 4.4. Statistical Analysis

Data were analyzed by one-way analysis of variance with a confidence level of 95% and *p* < 0.05 considered as statistical difference. Analyzed data were presented as means ± SD obtained from three independent experiments.

## 5. Conclusions

In the present study, it is hypothesized that the scaffolds made from low MW collagen and alginate could possess high bioactivity and flexible structure that could have close contact with the wound sites and could lessen discomfort such as hurt feelings when the scaffolds are applied on patients. Indeed, the prepared scaffolds showed a highly soft structure and promising bioactivity including antioxidant and coagulant activities; therefore, the results meet the goals of the study. Although there was no significant difference in antioxidant capacity between scaffolds, there were trends that, first, scaffolds containing low MW collagen (C6-A250) improved free radical scavenging activity as compared with those containing high MW collagen (C25-A250), and second, scaffolds containing modified alginate (C25-A125, C25-A63, and C25-A21) tended to increase radical scavenging activity when the treating temperature increased. In another word, the antioxidant capacity of scaffolds tends to increase with the decrease in the MW of biopolymers due to more functional groups such as hydroxyl, carbonyl, and carboxyl groups that contain more electron donors are produced. Scaffolds containing low MW alginate (C25-A63, C25-A21) exhibited significant coagulant capacity as compared with those containing high MW alginate (C6-A250, C25-A250, C25-A125). However, the difference in MW of collagen did not significantly influence on the coagulant activity (C6-A250 and C25-A250).

All the scaffolds possessed a highly porous structure with approximately 93%, thus they meet the requirement of the desired scaffold. Besides, the prepared scaffolds, except C25-A63, showed a high cross-linking degree with greater than 50%. Consequently, these scaffolds exhibited a low weight loss and high water-retention capacity, especially C25-A63. On the other hand, C6-A250 had poor physicochemical stability with the highest weight loss and enzymatic degradation, hence, this scaffold might have no practical applications. For further studies, the improvement of physicochemical stability of these scaffolds using bio-conjugation with phenolic compounds should be considered.

## Figures and Tables

**Table 1:** Molecular weights of collagen and alginate.

Materials	Average Molecular Weight (Da)	Abbreviation
Collagen 1	~6000	C6
Collagen 2	25,000	C25
Untreated alginate	32,000–250,000	A250
Alginate hydrolyzed at 110 °C	125,000	A125
Alginate hydrolyzed at 120 °C	63,000	A63
Alginate hydrolyzed at 130 °C	21,000	A21

**Table 2:** Peak positions of FTIR spectra of pure materials and scaffolds (“-“—not applicable).

Type of Fabrication	Absorption Band Peaks (cm^−1^)					
	Amide A	Amide B	Amide I	Amide II	Amide III	C‒O‒C
Collagen	3282	3057	1627	1525	1236	-
Sodium alginate	-	-	-	-	-	1024
C6-A250	3301	3074	1635	1542	1240	1028
C25-A250	3301	3095	1638	1540	1240	1024
C25-A125	3295	3082	1633	1540	1239	1029
C25-A63	3301	3071	1636	1540	1237	1026
C25-A21	3289	3068	1639	1546	1237	1027

## Data Availability

Not applicable.
